# Promoting the integrated community case management of pneumonia in children under 5 years in Nigeria through the proprietary and patent medicine vendors: a cost-effectiveness analysis

**DOI:** 10.1186/s12962-021-00265-9

**Published:** 2021-02-25

**Authors:** Charles E. Okafor, Obinna I. Ekwunife, Sabina O. Nduaguba

**Affiliations:** 1 Centre for Applied Health Economics, School of Medicine, Griffith University, 170 Kessels Road, Nathan, QLD 4111 Australia; 2 Menzies Health Institute, Southport, QLD Australia; 3 Department of Clinical Pharmacy and Pharmacy Management, Nnamdi Azikiwe University, Awka, Anambra Nigeria; 4 Pharmaceutical Outcomes and Policy Department, University of Florida College of Pharmacy, Gainesville, FL USA; 5 Center for Drug Effectiveness and Safety, University of Florida, Gainesville, FL USA

**Keywords:** Proprietary and patent medicine vendors, Amoxicillin dispersible tablets, Benefit, Education, Support, Nigeria

## Abstract

**Background:**

While evidence-based recommendations for the management pneumonia in under-5-year-olds at the community level with amoxicillin dispersible tablets (DT) were made by the World Health Organisation, initiatives to promote the integrated community case management (iCCM) of pneumonia through the proprietary and patent medicine vendors (PPMVs) have been poorly utilized in Nigeria, possibly due to low financial support and perceived benefit. This study provides costs, benefits and cost-effectiveness estimates and implications of promoting the iCCM through the PPMVs’ education and support. The outcome of this study will help inform healthcare decisions in Nigeria.

**Methods:**

This study was a cost-effectiveness analysis using a simulation-based Markov model. Two approaches were compared, the ‘no promotion’ and the ‘promotion’ scenarios. The health outcomes include disability-adjusted life years averted and severe pneumonia hospitalisation cost averted. The costs were expressed in 2019 US dollars.

**Results:**

The promotion of iCCM through the PPMVs was very cost effective with an incremental cost-effectiveness ratio of US$143.77 (95% CI US$137.42–150.50)/DALY averted. The promotion will prevent 28,359 cases of severe pneumonia hospitalisation with an estimated healthcare cost of US$390,578. It will also avert 900 deaths in a year.

**Conclusion:**

Promoting the iCCM for the treatment of pneumonia in children under 5 years through education and support of the PPMVs holds promise to harness the benefits of amoxicillin DT and provide a high return on investment. A nationwide promotion exercise should be considered especially in remote areas of the country.

## Background

The World Health Organisation (WHO) recommends the use of oral amoxicillin dispersible tablet (DT) as the first-line agent in the management of uncomplicated cases of pneumonia in children under 5 years at the community level and parenteral penicillin and gentamicin as first-line in severe cases [1], but implementation in Nigeria remains poor especially in rural communities.

The Nigerian Ministry of Health in collaboration with the Maternal and Child Survival Program (MCSP), and the WHO have implemented the Saving One Million Lives project [2]. This project aims to support community case management of illness such as pneumonia, malaria and diarrhoea as a strategy to improve treatment access and coverage especially for children living in rural areas [3]. The integrated community case management (iCCM) strategy enables assessment, classification, treatment and referral of pneumonia, diarrhoea and malaria cases [4]. The United Nations agencies and other donor agencies have supported the implementation of the iCCM in Nigeria [2]. Following the implementation of the national iCCM guidelines in the year 2013, the occurrence of childhood illness has reduced, and management of pneumonia improved but implementation remains poor [2]. A plausible reason for the poor result was the initial focus of iCCM support on community health workers and less focus on the proprietary and patent medicine vendors (PPMVs) who are ubiquitous in the communities. The PPMVs are drug vendors without formal training in pharmacy and are issued a licence by the Pharmacist Council of Nigeria (PCN) to retail non-prescription medications. The iCCM strategy has led to the removal of amoxicillin DT from the prescription-only medicine list to enable the PPMVs to have access to the drug for the treatment of non-severe childhood pneumonia at the community level [5]. In Nigeria, this decision was also supported by the fact that majority of the populace in rural areas visit PPMVs for medical advice, diagnosis, medications and general health management due to their low service cost and accessibility [6, 7]. A study in Uganda has also shown that the PPMVs are indispensable at the rural communities [8]. The study showed that promoting iCCM for uncomplicated pneumonia through the support of PPMVs has the potential to reduce the disease burden [8]. The coalition promoting the iCCM foresaw a problem of abuse and increased resistance to amoxicillin DT due to the possibility of irrational dispensing. This foreseen challenge called for the need to educate PPMVs (due to their low medical knowledge) who will sell the amoxicillin DT to patients. The training of PPMVs involves basic education about signs of pneumonia including danger signs, use of respiratory rate timers, how to dose the drug and when to refer patients in complicated cases to healthcare facilities.

The MCSP in collaboration with the United States Agency for International Development (USAID) conducted an education outreach in the year 2017 in four local government areas (Idah, Okehi, Izzi and Ohaozara) of two states in Nigeria (Kogi state and Ebonyi state) to test the feasibility of promoting the iCCM through the PPPVs [9]. The training held also included education on management of diarrhoea and malaria at the community level and when to refer patients to healthcare facilities [9]. In addition to supporting one of the training in Kogi state, Nemel Pharmaceuticals Limited, an indigenous pharmaceutical company of Nigeria in the year 2018 sponsored the iCCM training through the PCN in few local government areas of Enugu state and the Federal Capital Territory of Nigeria [9]. The outreach reported over 90% turn out of the expected trainees. Following the successful implementation of the training, the PPMVs national executive committee demanded promotion in other PPMVs locations. However, the promotion was decelerated possibly due to fund limitation and low perceived benefit [9]. This lacuna calls for evidence-based analysis of its benefit to encourage government and donor agencies buy-in. This problem has informed the need for health economic evaluation of the promotion exercise to assess whether scale-up of education and support of the PPMVs in the practice of iCCM for under-five pneumonia management will be beneficial to Nigeria.

This study, therefore, aimed to evaluate the cost-effectiveness of promoting iCCM for the management of pneumonia for children under 5 years in Nigeria through education and support of the PPMVs.

## Methods

### Study setting and sample size

The PPMV shops and community pharmacies are the major providers of health service at the community level for non-severe health conditions in Nigeria. The survey by the MCSP and the USAID showed that care-seeking at PPMVs shops for fever, diarrhoea, cough or pneumonia was about 40% in Nigeria and was used in this study [9]. Based on the care-seeking, the population used in the study was 40% of 34.6 million Nigerian children under 5 years based on the 2019 population report and the care-seeking at PPMV shops [10]. This population was used as the starting population in the model (well state) but at risk of having childhood pneumonia.

### Comparators

A. No promotion: In this scenario (current scenario), no treatment or wrong treatment is given to patients with non-severe pneumonia (moderate pneumonia) due to poor knowledge about the diagnosis and management by the PPMVs.
B. Promotion: In this scenario, due to the knowledge and support received, the PPMVs can rightly diagnose and treat non-severe pneumonia cases with amoxicillin DT for 5 days. This scenario involves the training of the PPMVs on signs of pneumonia including danger signs, use of respiratory rate timers, how to dose the drug and when to refer patients in complicated cases to healthcare facilities. It also includes the free distribution of one respiratory rate timer to each PPMV shop.

### Choice of model and assumptions

**Fig. 1:**
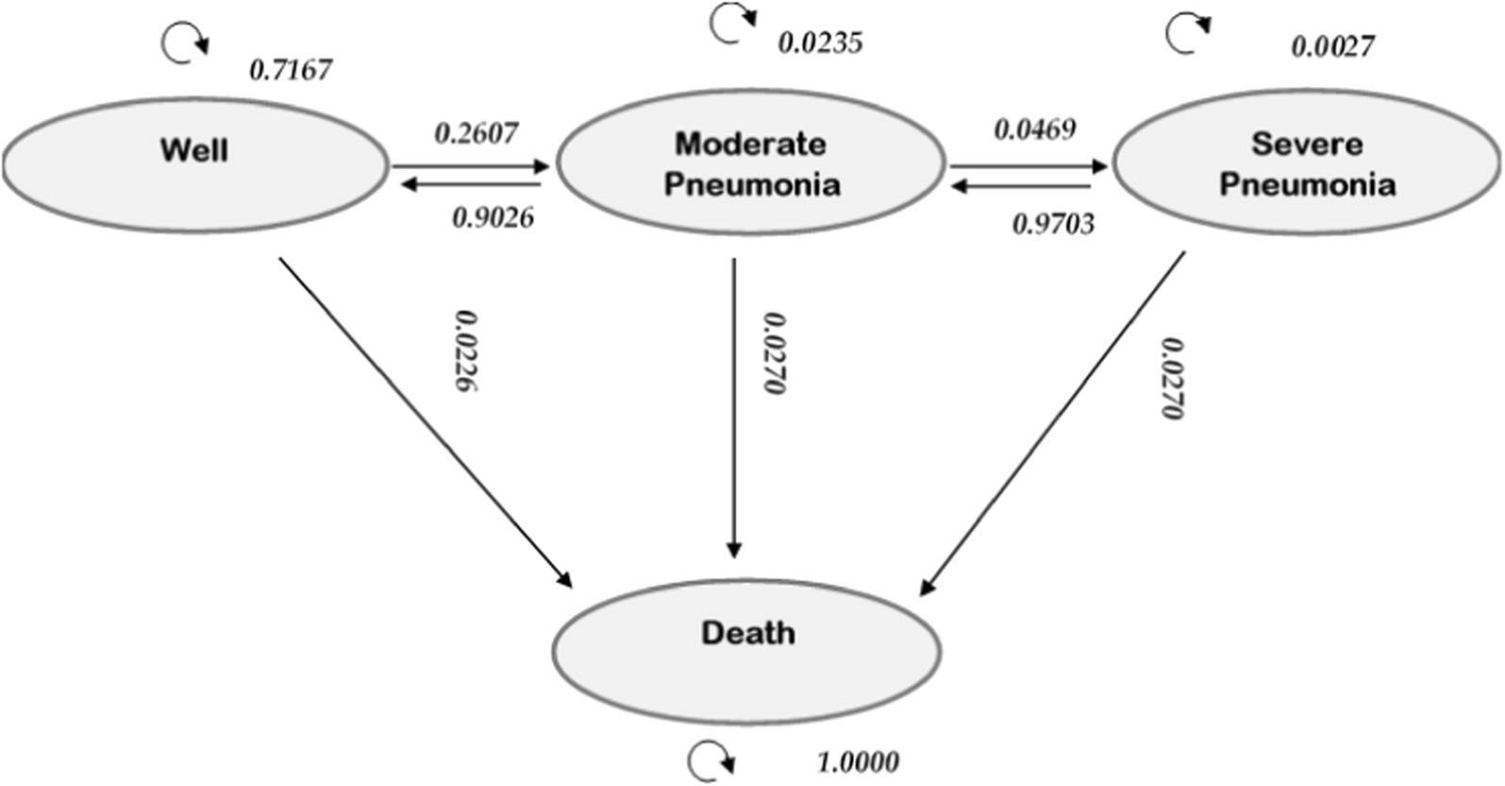
Model of the health states and the transition probabilities. The circles indicate the health states; the straight arrows indicate the possibility of transition to the different health states; the curly arrows indicate the possibility of remaining in the different health states

We used a simulation-based Markov model in our analysis with a yearly cycle for 5 years. The states in the model were: well state, moderate (clinical) pneumonia, severe pneumonia and death [11, 12]. The starting age in the model was under-1 year. The model was developed using retrospective data for Nigeria. The transition probabilities of moving to moderate and severe states were estimated from the national incidence rates from a global systematic review [1, 11]. Yearly incidence rates were converted to yearly probabilities using the formula, P = 1—${e}^{(-rt)}$ where P = probability, r = rate and t = time (year). The mortality rate from all-cause of disease and from pneumonia were obtained from the 2019 Institute of Health Metrics and Evaluation report for Nigeria [13]. In the model, children will start from the well state and with each cycle, they may remain well, move to moderate or severe pneumonia or may die as shown in Fig. 1.

### Time horizon and discount rate

The model simulated cost and outcome within the period of 0 to 4 years for the target population at risk of having pneumonia. The cost and effectiveness (health outcome) were discounted at a rate of 5% [14].

### Determination of cost

Based on the recent education programs to PPMVs conducted by the MCSP, the USAID and the PCN described earlier and elsewhere [9], we estimated that one health educator will train 10 PPMVs per day (in two sessions); 4 training sessions (2 days) will be held per week per health educator; 16 training sessions will be held per month per health educator and 176 training sessions will be held per annum (11 months) per health educator. The twelfth month (which varies across personnel) will serve for their leave of absence. The experience from the recent outreach revealed that at least a day-spacing is necessary for trainers to prepare for and travel to the next training venue. We assumed that for each PPMV shop, one representative will be trained. The number of PPMV shops was obtained from a recent survey in Nigeria as 24.58 per 100,000 population [15]. Based on the 2019 Nigerian population of about 201 million, 56 health educators will be required [10]. We included two extra educators in case of unforeseen shortfall of educators which brings the total to 58 educators. Based on Nigeria’s 6 geopolitical zones, 8 educators were assigned to each of four geopolitical zones (north-central; northeast; south-east; and south-south). A total of 12 and 11 educators were assigned to north-west and south-west zone respectively due to their relatively high population with north-west having the highest in Nigeria. One educator will be stationed in the northern region in case of any surge or demand while the other in the southern region. Three vehicles and three electronic projectors will be allocated to each geopolitical zone, while each trainer allocated a computer laptop. The outreach in 2018 revealed that at least five educators were needed at each training location to carter for the number of the trainees. This implies that there will be a maximum of two locations of training per zone per day. Details of the assumptions are shown in Table 1 and Additional file 1.

**Table 1:** Input data and assumptions in the model

Assumptions	Quantity	Uncertainty	Source
PPMVs and educators			
Number of PPMV shops per 100,000 population	24.58	(± 10%)	[15]
Estimated number of PPMV shops in Nigeria	49,397	(± 10%)	[15]
The ratio of PPMVs to health educators	10:1	N/A	Year 2018 outreach
Number of trainings per week per educator	4	N/A	Year 2018 outreach
Trainings per educator per annum	176	(± 10%)	Year 2018 outreach
Health educators needed (2 additional)	58	(± 10%)	Year 2018 outreach
The average number of trainees per meeting	80	(50–100)	Year 2018 outreach
Number of training auditoriums	617	(± 10%)	Year 2018 outreach
Training refreshments	53,162	(± 10%)	Year 2018 outreach
Care-seeking at PPMV shops	40%	(± 10%)	[9]
Overall care-seeking at health facilities	35%	(± 10%)	[2, 9]
Number of respiratory rate timers	49,397	(± 10%)	[15]
Administrative requirements			
Program director	1	N/A	[16]
Program coordinator	1	N/A	[16]
Administrative officer	1	N/A	[16]
Office and space (1000 m square)	1	N/A	[16]
Data entry clerk	1	N/A	[16]
Finance officer	1	N/A	[16]
Health educators/Trainers	58	(± 10%)	[16]
External consultant (12 consultations)	1	N/A	[16]
Logistics/social worker	1	N/A	[16]
Transportation drivers	20	N/A	[16]
Vehicles	20	N/A	[16]
Useful years of each vehicle	8	N/A	[16]
Electronic projectors for training	19	N/A	Year 2018 outreach
Computers and Laptops	61	(± 10%)	Year 2018 outreach
Useful years of computers and projectors	1	N/A	–
Transport allowance (frequency)	602	(± 10%)	Year 2018 outreach
Electricity utility (KWH)	12,000	(± 10%)	[17]
Telephone utility (closed user group)	70	(± 10%)	Local tariff
Number of hotel accommodations	50,104	(± 10%)	Year 2018 outreach

*PPMV* Proprietary and patent medicine vendors, *KWH* kilowatts hour

The costs were estimated from the payers’ perspective (government, donor agencies or third-party payer). The cost of amoxicillin DT was not captured in the model because patients’ caregivers will pay out-of-pocket. Thus, only the incremental cost (cost of promotion was considered in the model). The total cost of the promotion includes several components. Some cost components of the promotion were obtained from WHO-CHOICE [16] for AFRO D region, while some were obtained from local survey. The human resource cost component which includes a program director, a program coordinator, health educators, an administrative officer, a data entry clerk, a finance officer, vehicle drivers, a logistic officer and an external consultant were obtained from WHO-CHOICE. The capital cost component which includes vehicles, office space, electronic projectors and computers were obtained from the local markets’ prices by one of the authors, OE, and from WHO-CHOICE. The cost of consumables which include electrical utility was obtained from the Nigerian electricity regulatory commission rate [17], while the cost of telephone calls was estimated based on the local survey. Welfare costs including hotel accommodation and travel allowance, were estimated based on the recent education outreach conducted in 2018 in Nigeria and from local survey. The cost data from the WHO-CHOICE were in 2005 international dollars, so, were converted to USD using the 2005 price level ratio of 0.345 for Naira to US dollar, which were then inflated to 2019 US dollars using the Gross domestic product (GDP) deflator index. The cost data obtained from local prices were converted to 2019 USD using an exchange rate of 307 Naira to a US dollar. The costs used in the model were annual costs since the model runs in a yearly cycle. The cost of vehicles was annualised based on useful life years of 8 years [16], while 1 year useful life year was used for the electronics. For each cost component, the total cost was estimated as product of the unit cost and the quantity described in Table 1. The cost of promotion was distributed across the under-5-year-olds based on the caregivers’ PPMV care-seeking of 40%. Gamma distribution was used to capture the uncertainty inherent in the cost parameters. Details of the costs are shown in Table 2 and Additional file 1.

**Table 2:** Parameters and distribution in the Markov model

Variable	Mean	Distribution (95% CI)	Source
Transition probabilities			
Well to moderate pneumonia	0.2607	Beta (0.1479–0.4161)	[1, 11]
Moderate pneumonia to severe pneumonia	0.0469	Beta (0.0178–0.1068)	[1, 11]
Recurrent moderate pneumonia	0.0235	Beta (0.0133–0.0375)	[1, 11, 22]
Recurrent severe pneumonia	0.0027	Beta (0.0010–0.0061)	[1, 11, 22]
Moderate pneumonia to well	0.9026	Beta (0.8106–0.9456)	[1, 11, 22]
Severe pneumonia to moderate pneumonia	0.9703	Beta (0.9598–0.9752)	[1, 11, 22]
Remaining well	0.7167	Beta (0.6878–0.8792)	[1, 11, 22]
All-cause mortality	0.0226	Beta (0.0186–0.0275)	[13]
Pneumonia to death	0.0270	Beta (0.0219–0.0314)	[13]
Relative risk of oral amoxicillin	0.485	Log-normal (0.372–0.731)	[18, 19]
Impact of the promotion	43.38%	N/A	[8]
Costs (US$)			
Program director	18,333.78	N/A (± 25%)	[16]
Program coordinator	12,213.40	N/A (± 25%)	[16]
Administration officer	7543.48	N/A (± 25%)	[16]
Office and space (1000 m square)	9422.51	N/A (± 25%)	[16]
Data entry clerk	5713.69	N/A (± 25%)	[16]
Finance officer	5713.69	N/A (± 25%)	[16]
Health educators/Trainers	43,7521.90	N/A (± 25%)	[16]
External consultant	555.69	N/A (± 25%)	[16]
Logistics/social worker	7543.48	N/A (± 25%)	[16]
Transportation drivers	87,635.44	N/A (± 25%)	[16]
Vehicles and maintenance (annualized)	142,050	N/A (± 25%)	[16]
Electronic projectors for training	6188.93	N/A (± 25%)	Year 2018 survey
Computers and Laptops	23,843.65	N/A (± 25%)	Year 2018 survey
Transport allowance	36,679.93	N/A (± 25%)	[16]
Electricity utility (KWH) @ $0.066/kwh	792.00	N/A (± 25%)	[17]
Telephone utility (closed user group)	2736.16	N/A (± 25%)	Local tariff
Hotel accommodation	202,133.00	N/A (± 25%)	Year 2018 survey
Respiratory diagnostic timer	2,173,468.00	N/A (± 25%)	[23]
Miscellaneous	12,000	N/A (± 25%)	–
Cost of promotion per child	0.25	Gamma (0.22–0.30)	Model
Disability weights			
Moderate (clinical) pneumonia	0.051	Beta (0.032–0.074)	[13]
Severe pneumonia	0.133	Beta (0.088–0.190)	[13]
Discount rate			
Cost	5%	N/A (min 0%, max 10%)	[14]
Utility	5%	N/A (min 0%, max 10%)	[14]

*KWH* kilowatts hours

### The measure of effectiveness (as relative risk ratio)

The effectiveness of oral amoxicillin was estimated from a multi-centre randomized, open-label trial [18] and a study on antimicrobial susceptibility pattern of invasive pneumococcal isolates in Nigeria [19]. The impact of iCCM promotion for pneumonia management through the PPMVs was obtained from a recent study in Uganda that compared the effect of promotion (through PPMVs education and support with a diagnostic kit and amoxicillin DT) to ‘no promotion’ based on the difference in the proportion and appropriateness of treated patients [8]. The difference in the use of amoxicillin DT and respiratory timer between the promotion and the non-promotion group was 49.3% and the appropriateness of care was 88% [8]. Details of the parameters inputs and distribution are shown in Table 2.

### Health outcome

The primary outcome of the promotion was measured as disability-adjusted life years (DALY) averted. The DALY calculation was based on 2019 Global Burden of Disease study parameters and we used recently updated disability weights for moderate and severe pneumonia [13]. The DALY was calculated as the sum of the years of life lived with disability (YLD) from morbidity and the years of life lost (YLL) from mortality for each cycle year. The YLD = number of cases × duration till remission or death × disability weight [13]. YLL = number of deaths due to pneumonia × life expectancy at the age of death [20]. The standard life expectancy of < 1 year (54.7) and 1–4 years of (57.9) was obtained from the Nigerian life table [21]. The DALY averted was calculated as the difference between the DALYs lost in the current scenario (no promotion) and the promotion scenario. The DALY was calculated for each cycle and accumulated over the time horizon of 5 years and averaged to obtain the mean DALY. The secondary outcome was measured as the cost of hospitalisation due to severe pneumonia averted.

### Analysis of data

The cost data, transition probabilities, relative risk and utilities were made probabilistic using the appropriate distributions as shown in Table 2. Half cycle correction using the life table method was employed in the model [24]. Incremental cost-effectiveness ratio (ICER) was the economic evaluation measure used in the assessment. This was estimated as the ratio of the incremental cost (cost of the promotion) to the DALY averted. A conservative cost-effectiveness threshold of 0.52 times the GDP per capita (US$ 2230) of Nigeria was used in the assessment [25]. A probabilistic sensitivity analysis (PSA) was used to assess simultaneous uncertainty in the variables. We ran 1000 iterations of Monte Carlo simulation to assess the sensitivity of the results to the parameters changes. A measure of the proportion of the simulations below the ICER threshold was estimated. The results from the simulation output of the PSA were presented as interval estimates (percentile intervals), in a cost-effectiveness plane and a cost-effectiveness acceptability curve. Furthermore, we performed a deterministic sensitivity of our assumptions on the outcome using a conservative approach of sensitivity analysis by increasing the cost of promotion by 25%, 50%, 75% and 100% (2 times increase). Data were analysed using Microsoft 365 Excel.

## Results

### Costs and outcomes

From the PSA, the estimated cost of promoting the iCCM through the PPMVs was US$3.46 (95% CI US$3.41–3.51) million for a 90% and above promotion coverage. The total DALYs averted due to the impact of the promotion was 24,061.17 (95% CI 23,332.12–24,790.23) DALYs. The ICER of the promotion was US$143.77 (95% CI US$137.42–150.50)/DALY averted. The results showed that the averted cases of severe pneumonia following the promotion approach was 28,359 cases which translates to an averted hospitalisation cost of about US$390,578 based on 35% care-seeking at health facilities and following the WHO and the paediatrician association of Nigeria treatment guideline [26]. The result also showed that the respiratory rate timers contributed to about 60% (US$2.17 million) of the total promotion cost. Table 3 presents details of the results.

**Table 3:** Costs and outcomes from the probabilistic sensitivity analysis

Costs and outcomes	Mean (95% confidence interval)
Cost of promotion (US$)	3,459,154 (3,406,782–3,511,527)
DALY without promotion	888,657 (855,752–921,564)
DALY with promotion	864,596 (832,625–896,568)
DALY averted	24,061 (23,332–24,790)
Deaths averted	900 (873–927)
Incremental cost-effectiveness ratio (US$/DALY averted)	143.77 (137.42–150.50)
Cases of severe pneumonia hospitalisation averted	28,359 (27,370–29,349)
Cost of severe pneumonia hospitalisation averted (US$)	390,578 (252,041–508,198)

**Fig. 2:**
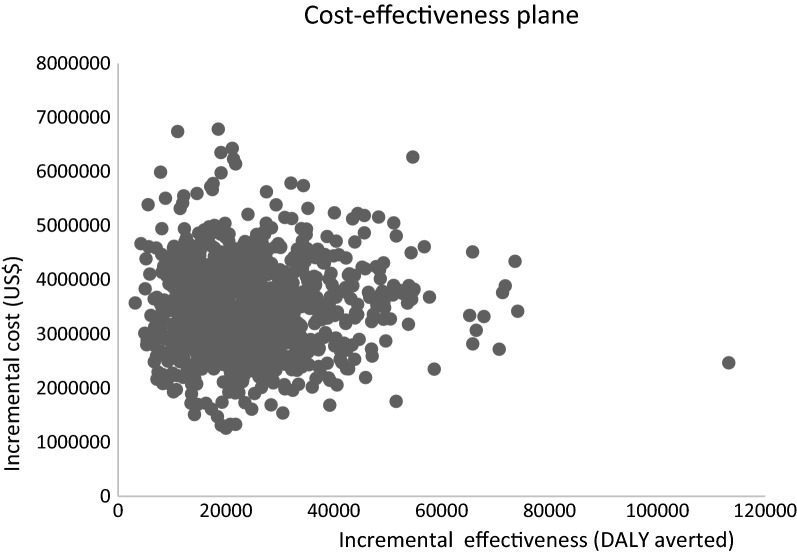
Cost-effectiveness plane of the promotion program

**Fig. 3:**
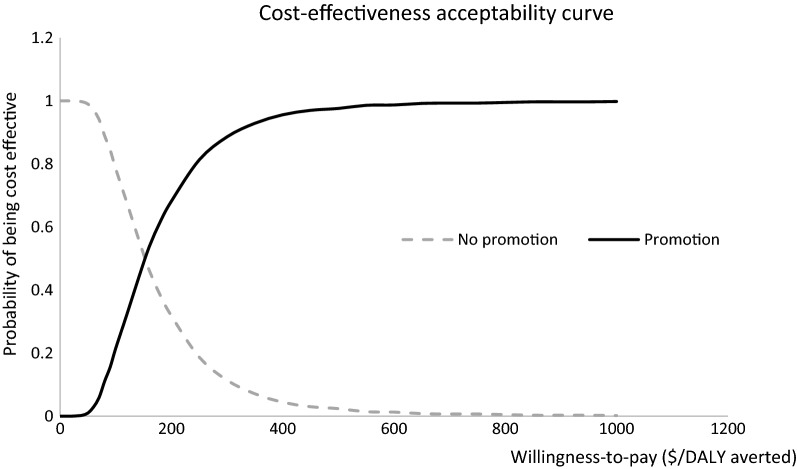
Cost-effectiveness acceptability curve of the promotion program at different willingness-to-pay thresholds

The PSA showed that the ICER result was not sensitive to the parameters. All the simulations (100%) were below the cost-effectiveness threshold of US$1160/DALY averted. Figure 2 presents the cost-effectiveness plane from the PSA, while Fig. 3 presents the cost-effectiveness acceptability curve showing the probability of the promotion being cost effective at different willingness-to-pay thresholds.

The deterministic sensitivity test also showed that the result was robust. The ICERs obtained were all below the cost-effectiveness threshold. The ICER (US$1,150/DALY averted) was also below the threshold when the promotion cost was increased up to 8 times the estimated cost. See Additional file 1 for details.

## Discussion

To the best of our knowledge, this study is the first to conduct an economic evaluation of promoting iCCM in the management of uncomplicated pneumonia through the PPMVs support. While evidence-based recommendations for the management of pneumonia in under-5-year-olds were made by the WHO, the implementation remains poor in Nigeria. Initiatives to promote the iCCM through the PPMVs has been poorly utilized possibly due to low financial support and perceived benefit. No study has quantified its cost and benefit in Nigeria, a region with high pneumonia incidence and mortality. This study provided costs, benefits and cost-effectiveness estimates and implications that will guide decision making in the country. The study showed that the iCCM promotion through the PPMVs using the approach described will be highly cost effective in Nigeria with an ICER of US$143.77 (95% CI US$137.42–150.50)/DALY averted. The ICER was about 15 times below the GDP per capita of Nigeria. Thus, implementing the promotion program should be considered.

The PPMV education outreach conducted in 2017 and 2018 incorporated education for malaria and diarrhoea management also. Thus, no additional resources need to be invested to provide education for malaria and diarrhoea management to the PPMVs. This implies that the actual cost for promoting under-five pneumonia management can become approximately one-third of the estimated amount in this study which will save cost.

Late diagnosis of childhood pneumonia is a major cause of pneumonia death in developing countries. The promotion exercise will lead to early identification of pneumonia cases and immediate treatment which will minimize severe cases, and pneumonia death. Due to the importance of continuous education, a biennial training exercise could be considered also.

There is also need for price and quality regulation of the amoxicillin DT supplied to the PPMVs. Regulatory measures to ensure that high quality products are supplied to the PPMVs at a regulated price will further guarantee the success of the iCCM promotion through the PPMVs.

This study has some limitations. First, we used data from a study in Uganda to apply the impact of the promotion program in Nigeria because it is the only study that have measured the impact of the program in Africa at the time of this study. The impact might vary during implementation in Nigeria. Second, the assumptions in estimating the cost of promoting iCCM through the PPMVs might also vary if implementated. The study estimates and assumptions were based on the recent outreach conducted in Nigeria. The sensitivity analysis however, showed that at a higher cost (up to 8 times the estimated cost), the promotion program will remain cost effective in Nigeria. Third, our estimate did not capture alternate treatment cost in case of the first-line failure. It also failed to capture the treatment approach for human immunodeficiency virus (HIV) patients with pneumonia or patients at risk of HIV [1, 26].

Nigeria is the giant of Africa, but the country has lost so many human resources and funds due to lack of information and right implementation practices. Most useful healthcare implementations are poorly sustained. The high mortality rate of children under 5 years in the country calls for a pragmatic approach to ameliorate the problem. Being the “future of tomorrow”, protecting the Nigerian children under 5 years should not be negotiated, otherwise, the frontier of ‘gigantism and dwarfism of Africa’ for Nigeria will become narrower.

## Conclusion

The use of amoxicillin DT is very cost effective and beneficial to Nigeria in cases of non-severe pneumonia, but its benefits have been poorly harnessed. Promotion of iCCM for the treatment of pneumonia in children under 5 years through education and support of the PPMVs holds promises to harness these benefits and additional benefits to the economy, providing a high return on investment. A nationwide promotion exercise should be considered especially in remote areas of the country.

## Supplementary Information

**Additional file 1.** Data analysis.

## Data Availability

Data used in the study are provided in the additional file.

## Abbreviations

DT: Dispersible tablets
MCSP: Maternal and child survival program
iCCM: Integrated community case management
PPMV: Proprietary and patent medicine vendors
PCN: Pharmacists council of Nigeria
USAID: United States agency for international development
YLD: Years lived with disability
YLL: Years of life lost
DALY: Disability-adjusted life years
